# Blood-Based Biomarkers for Improved Characterization of Traumatic Brain Injury: Recommendations from the 2024 National Institute for Neurological Disorders and Stroke Traumatic Brain Injury Classification and Nomenclature Initiative Blood-Based Biomarkers Working Group

**DOI:** 10.1089/neu.2024.0581

**Published:** 2025-07-09

**Authors:** Jeffrey J. Bazarian, Henrik Zetterberg, András Buki, Bradley A. Dengler, Ramon Diaz-Arrastia, Frederick K. Korley, Rachel Lazarus, Timothy B. Meier, Stefania Mondello, Kasey Moritz, David O. Okonkwo, Linda Papa, James B. Phillips, Jussi P. Posti, Ava M. Puccio, Stephanie Sloley, Ewout Steyerberg, Kevin K. Wang, Hibah O. Awwad, Kristen Dams-O’Connor, Adele Doperalski, Andrew I.R. Maas, Michael A. McCrea, Nsini Umoh, Geoffrey T. Manley

**Affiliations:** ^1^ Departments of Emergency Medicine and Neurology, University of Rochester School of Medicine and Dentistry, Rochester, New York, USA.; ^2^ University of Gothenburg, Gothenburg, Sweden.; ^3^ Örebro University, Örebro, Sweden.; ^4^ Military Traumatic Brain Injury Initiative, Uniformed Services University of the Health Sciences, Bethesda, Maryland, USA.; ^5^ Department of Neurology, University of Pennsylvania, Philadelphia, Pennsylvania, USA.; ^6^ Department of Emergency Medicine, University of Michigan, Ann Arbor, Michigan, USA.; ^7^ American Association of Retired Persons, Washington District of Columbia, USA.; ^8^ Department of Neurosurgery, Medical College of Wisconsin, Milwaukee, Wisconsin, USA.; ^9^ University of Messina, Messina, Italy.; ^10^ U.S. Army Medical Research and Development Command, Combat Casualty Care Research Program, Fort Detrick, Maryland, USA.; ^11^ Department of Neurological Surgery, University of Pittsburgh, Pittsburgh, Pennsylvania, USA.; ^12^ Orlando Health Orlando Regional Medical Center, Orlando, Florida, USA.; ^13^ Neurocenter, Department of Neurosurgery and Turku Brain Injury Center, Turku University Hospital and University of Turku, Turku, Finland.; ^14^ TBI Center of Excellence, Defense Health Agency, Silver Spring, Maryland, USA.; ^15^ University of Leiden, Leiden, Netherlands.; ^16^ Center for Neurotrauma, Multiomics & Biomarkers, Neuroscience institute, Morehouse School of Medicine, Atlanta, Georgia, USA.; ^17^ Center for Visual and Neurocognitive Rehabilitation, Atlanta VA Health Care System, Decatur, Georgia, USA.; ^18^ Division of Neuroscience, National Institute of Neurological Disorders and Stroke, Bethesda, Maryland, USA.; ^19^ Department of Rehabilitation and Human Performance, Icahn School of Medicine, Mount Sinai, New York, New York, USA.; ^20^ Department of Neurology, Icahn School of Medicine, Mount Sinai, New York, New York, USA.; ^21^ Department of Neurosurgery, Antwerp University Hospital, Edegem, Belgium.; ^22^ Department of Translational Neuroscience, Faculty of Medicine and Health Science, University of Antwerp, Antwerp, Belgium.; ^23^ Neurological Surgery, University of California San Francisco, San Francisco, California, USA.

**Keywords:** brain biomarkers, concussion, glial fibrillary acidic protein, neurofilament light chain, S100B calcium-binding protein, traumatic brain injury, ubiquitin C-terminal hydrolase L1

## Abstract

A 2022 report by the National Academies of Sciences, Engineering, and Medicine called for a Traumatic Brain Injury (TBI) Classification Workshop by the National Institutes of Health (NIH) to develop a more precise, evidence-based classification system. The workshop aimed to revise the Glasgow Coma Scale-based system by incorporating neuroimaging and validated blood biomarker tests. In December 2022, the National Institute for Neurological Disorders and Stroke formed six working groups of TBI experts to make recommendations for this revision. This report presents the findings and recommendations from the blood-based biomarker (BBM) working group, including feedback from the workshop and subsequent public review. The application of BBMs in a TBI classification system has potential to allow for a more adaptable and nuanced approach to triage, diagnosis, prognosis, and treatment. Current evidence supports the use of glial fibrillary acidic protein (GFAP), ubiquitin C-terminal hydrolase L1, and S100B calcium-binding protein (S100B) to assist in reclassification of TBI at acute time points (0–24 h) primarily in emergency department settings, while neurofilament light chain (NfL), GFAP, and S100B have utility at subacute time points (1–30 days) in-hospital and intensive care unit settings. Blood levels of these biomarkers reflect the extent of structural brain injury in TBI and may be useful for describing the extent of structural brain injury in a classification system. While there is insufficient evidence to support a role for BBMs at chronic time points (>30 days), emerging evidence suggests that NfL and phosphorylated tau may have a potential future role in this regard. For inclusion in a revised TBI classification system, BBM assays must have appropriate age- and sex-specific reference ranges, be harmonized across platforms, and achieve high analytical precision, including accuracy, linearity, detection limits, selectivity, recovery, reproducibility, and stability. Improving transparency in BBM assay development can be achieved through large-scale data sharing of methods and results. Future research should focus on methods for promoting clinical adoption of BBM results, correlating BBMs with advanced neuroimaging, and on discovering new biomarkers for improved diagnosis and prognosis.

## Overview of Literature and Current State

The current classification system for traumatic brain injury (TBI) based on the 13-point Glasgow Coma Scale (GCS) and duration of loss of consciousness (LOC) and post-traumatic amnesia (PTA) is widely seen as imprecise.^1^ It categorizes TBI as mild (GCS 13–15, LOC <30 min, PTA <24 h), moderate (GCS 9–12, LOC and <1 week), or severe (GCS 3–8, LOC and PTA >1 week). Various factors, such as intubation, sedation, intoxication, and interrater differences, can affect GCS score determination as well as the assessment of duration of LOC and PTA. Additionally, the GCS’s ceiling effect limits its precision, as many patients with significant TBI can score 15. It also fails to provide insight into the underlying brain injury mechanisms (such as axonal injury, neuroinflammation, oxidative stress), hindering the development of targeted therapies, and has limited predictive value for post-TBI outcomes, complicating timely interventions.

A 2022 report by the National Academies of Sciences, Engineering, and Medicine^2^ called for a TBI Classification Workshop by the NIH to develop a more precise, evidence-based classification system. The workshop aimed to revise the GCS-based system by incorporating neuroimaging and validated blood biomarker tests. In December 2022, the National Institute for Neurological Disorders and Stroke (NINDS) formed six working groups of TBI experts to make recommendations for this revision. This report presents the findings and recommendations from the blood-based biomarker (BBM) working group, including feedback from the workshop and subsequent public review.

BBMs are emerging as valuable tools for evaluating and managing TBI by providing objective measures of injury severity, prognosis, and treatment response. They hold the potential to assist clinicians in early diagnosis, risk stratification, and patient monitoring, enabling more personalized treatment strategies. Reflecting underlying pathobiological processes like neuronal and vascular injury, BBMs also promise to aid in developing targeted therapies, leading to timely interventions and improved patient outcomes while reducing long-term complications.

Over the past two decades, BBMs have transitioned from research tools to clinical applications, assisted by NINDS/NIH efforts that standardized data collection and analysis.^3,4^ This progress has fostered large-scale TBI research consortia, such as Transforming Research and Clinical Knowledge in TBI (TRACK-TBI) in the United States^5^ and Collaborative European NeuroTrauma Effectiveness Research in TBI (CENTER-TBI) in Europe.^6^ A significant milestone was the 2013 integration of the brain astrocyte protein S100B into mild TBI diagnostics by the Scandinavian Neurotrauma Committee.^7^ This was followed by the FDA’s 2018 clearance of an assay combining glial fibrillary acidic protein (GFAP) and ubiquitin C-terminal hydrolase L1 (UCH-L1), based on the results of the Prospective Clinical Evaluation of Biomarkers of TBI (ALERT-TBI) trial funded by the US Department of Defense.^8^ The 2023 French TBI guidelines further enhanced clinical practice by incorporating S100B and GFAP-UCH-L1 into the diagnostic framework for mild TBI.^9^

The NINDS TBI Classification and Nomenclature Initiative BBM working group aimed to identify and characterize BBMs with sufficient analytical and clinical validity to support a new TBI classification system. The work group focused on biomarkers backed by substantial scientific evidence, significant diagnostic or prognostic utility, and robust commercially viable assays. The diagnostic utility of BBMs was described in the context of “aid in diagnosis,” which the Food and Drug Administration (FDA) defines as any device or tool that helps health care professionals identify or diagnose a disease or condition, but which is not intended to be the sole method of diagnosis.^10^ The group also identified BBMs ready for near-term integration and outlined research gaps and directions needed for implementing BBMs in the new TBI system, along with recommendations for their analysis and interpretation.

These recommendations were delineated for three distinct postinjury time epochs: acute (0–24 h), subacute (1–30 days), and chronic postinjury (beyond 1 month). Furthermore, the working group considered the clinical settings where BBMs could have an impact, such as emergency departments (EDs) and intensive care units (ICUs), as well as clinical variables that could potentially enhance diagnostic or prognostic utility.

## Methods

A narrative review approach was used to identify and summarize the evidence supporting the working group’s recommendations. The content expertise of working group members was leveraged to identify biomarkers with supporting evidence likely to meet the specific criteria mentioned above. Preference was given to primary research articles estimating BBM diagnostic and/or prognostic utility. The BBM working group was comprised of TBI content experts in neurology (R.D.A.), neurosurgery (A.B., D.O.O., J.P.P.), neuroscience (T.B.M., K.K.W., R.L., A.P.), clinical chemistry (H.Z.), military medicine (B.D., J.B.P., K.M., S.S.), critical care medicine/traumatology (S.M., J.P.P.), emergency medicine (J.J.B., F.K., L.P.), and biostatistics and medical decision-making (E.S.). Countries represented in the working group included Finland, Italy, Netherlands, Sweden, and the United States. The working group met regularly during 2023 to determine a strategy for addressing the overall objectives and to draft an initial set of recommendations. Data from these articles were compiled in tabular format to provide supporting evidence for the working group’s recommendations.

The initial draft of the BBM working group was circulated to members of the other five working groups for feedback, which was incorporated into a subsequent draft report. This report was posted on the NINDS website 2 weeks prior to an open meeting at NIH on January 22 and 23, 2024, during which BBM recommendations were presented and expert/peer group and public feedback was sought during the workshop and after the workshop. Feedback was incorporated into an updated report which was then reviewed by the Steering Committee. The BBM work group met again several times to discuss and integrate the feedback from the Steering Committee to create this final report.

### Measures of BBM accuracy used in this report

Many BBM studies use the area under the receiver operating characteristic curve (AUC) to characterize the ability of BBMs to discriminate between (1) those with and without TBI, (2) patients with TBI with and without CT-detected intracranial injury (ICI), and (3) patients with TBI with and without unfavorable or incomplete long-term functional outcome. The receiver operating characteristic (ROC) curve is a graph used to evaluate how well a test measured on a continuous scale, such as blood levels of a BBM, can distinguish between two groups. The curve plots the True Positive Rate (sensitivity) on the *y*-axis and the False Positive Rate (1-specificity) on the *x*-axis at various thresholds or “cutoffs” of BBM levels. The shape of the ROC curve and the area under it provide insight into the test’s overall accuracy. A curve closer to the top left corner (i.e., AUC closer to 1.0) indicates a test better able to distinguish between the two groups. A curve closer to the diagonal (i.e., AUC closer to 0.5) indicates that the test has no discriminatory power. An inherent property of the ROC curve is that there is a tradeoff between sensitivity and specificity as one moves along the various BBM cutoffs. At low cutoff values, sensitivity is high and specificity is low, at high cutoff values, the reverse is true. Researchers typically chose a cutoff to maximize either the sensitivity or the specificity, depending on the clinical objective.

## Key Findings

### Biomarkers with utility at acute time points (**<**24 h after injury)

Acutely measured blood levels of GFAP, UCH-L1, and S100B were found to have great potential to fill gaps in the current GCS-based TBI classification system because of their demonstrated utility as aids to diagnosis and prognosis in a range of acute care settings, from EDs to athletic training rooms. In addition, all three biomarkers provide a measure of TBI severity through their ability to accurately predict the absence of traumatic ICI on contemporaneously acquired head CT scans and have received regulatory approval from the US FDA or European Medicines Agency (EMA) for this indication (Table 1).

**Table 1.** Ability of FDA or CE-Cleared Assays for Glial Fibrillary Acidic Protein, Ubiquitin C-Terminal Hydrolase L1, and S100B to Predict Traumatic Intracranial Injury on Head CT Scan at Acute Time Points

Assay	Limits of quantification^*f*^	Time postinjury (h)	Cutoff^*f*^	Patient with TBI group (GCS)	Sensitivity (95% CI)	Specificity (95% CI)	NPV (95% CI)	PPV (95% CI)	Reference
GFAP+UCH-L1									
Banyan BTI [serum]	G: 10–320 U: 80–2560	0–12	G: 22 U: 327	9–15	0.976 (0.931–0.995)	0.364 (0.342–0.387)	0.996 (0.996–0.999)	0.092 (0.079–0.112)	Bazarian (2018)^8^
				14–15	0.973 (0.924–0.994)	0.367 (0.345–0.390)	0.995 (0.987–0.999)	0.088 (0.073–0.105)	
i-STAT Alinity TBI Plasma Test^a^ [EDTA plasma]	G: 30–10000 U: 200–3200	0–12	G: 30 U: 360	13–15	0.958 (0.906–0.982)	0.404 (0.382–0.427)	0.993 (0.985–0.997)	0.098 (0.082–0.116)	Bazarian (2021)^11^ US FDA (2024)^12^
		0–12	G: 30 U: 360	15	0.957 (0.896–0.983)	0.411 (0.387–0.434)	0.994 (0.985–0.998)	0.083 (0.068–0.098)	
		0–2	G: 30 U: 360	15	1.00 (0.723–1.00)	0.356 (0.304–0.412)	1.00 (0.965–1.00)	0.050 (0.027–0.089)	
i-STAT TBI^a^ [EDTA whole blood]	G: 47–10000 U: 87–3200	0–24	G: 65 U: 360		0.965	0.403	0.965 (adj: 0.994)	0.40 (adj: 0.094)	US FDA (2024)^13^
Alinity i/ARCH TBI^a^ [plasma and serum]	G: 6–42000 U: 26–25000	0–12	G: 35 U: 400	9–15	0.967 (0.917–0.987)	0.401 (0.378–0.424)	0.994 (0.986–0.998)	0.098 (0.082–0.116)	US FDA (2023)^14^
VIDAS® TBI (GFAP, UCH-L1)^b^ [serum]	G: 10–320 U: 80–2560	0–12	G: 22 U: 327	13–15	0.967 (0.917–0.991)	0.412 (0.389–0.435)	0.995 (0.986–0.999)	0.099 (0.083–0.118)	US FDA (2024)^15^
S100B									
Elecsys®S100^c^ [serum]	S: 0.005–39^d^ S: 0.015–30^e^	0–3	S: 0.105	13–15	0.988 (0.965–1.00)	0.329 (0.030–0.359)	0.997 (0.991–1.00)	0.11 (0.088–0.133)	Roche (2023)^16^

^a^ Abbott.

^b^ bioMerieux.

^c^ Roche.

^d^ Cobas e411, e601/e602.

^e^ Cobas e402, Cobas e801.

^f^ G: GFAP (pg/mL), U: UCH-L1 (pg/mL), S: S100B (μg/L).

BTI, brain trauma indicator; CI, confidence interval; GCS, glasgow coma scale; GFAP, glial fibrillary acidic protein; NPV, negative predictive value; PPV, positive predictive values; S100B, S100B calcium-binding protein; TBI, traumatic brain injury; UCH-L1, ubiquitin C-terminal hydrolase L1.

### Glial fibrillary acidic protein

GFAP is an intermediate filament protein found mainly in the cytoskeleton of astroglia but also in nonmyelinating Schwann cells and enteric glial cells.^17^ GFAP released into the blood following TBI is detectable within 30 min of injury, peaks within 20–24 h, declines gradually over 72 h (but is still detectable at 168 h), and has an apparent half-life of 24–48 h.^18–22^

#### Prediction of traumatic ICI on CT and MRI

Low or undetectable serum/plasma levels of GFAP within the initial hours of TBI have been extensively studied for their ability to identify individuals at very low risk for traumatic ICI on CT scans, potentially reducing unnecessary acute neuroimaging. There is a consensus that CT scans are often overused in low-risk patients, and many are avoidable.^23,24^ A 2017 systematic review highlighted 24 studies showing a positive correlation between GFAP levels and acute traumatic ICI on CT, with AUCs ranging from 0.74 to 0.98, indicating strong discriminatory power.^25^ The GFAP cutoffs for high sensitivity and negative predictive value (NPV) were primarily determined in studies combining it with UCH-L1. In these studies, elevations above the cutoffs of one or both markers indicated a positive test, while levels below the cutoffs for both markers defined a negative result. At GFAP cutoffs of 22–65 pg/mL, sensitivities for CT-detected ICI ranged from 0.96 to 1.00 and NPVs from 0.97 to 1.00^8,11–15^ (Table 1). However, specificities and positive predictive values (PPV) were more modest, between 0.36 and 0.41 and 0.05 and 0.10, respectively. In these studies, most subjects had GCS scores of 13–15, and biomarkers were measured within 12 h of injury. GFAP, in combination with UCH-L1, has received FDA and EMA clearance for predicting normal CT results within 24 h postinjury.^12–15^ Moreover, cutoffs for point-of-care test devices have been derived and validated.^11,26^ It is also recommended for use in French Mild TBI Practice Guidelines.^9^ Additionally, GFAP may help predict traumatic ICI visible only on MRI, showing an AUC of 0.78 (95% confidence interval [CI]: 0.73–0.83) for distinguishing those with and without abnormalities 7–18 days postinjury.^27^

#### Aid in diagnosis

Serum/plasma levels of GFAP have considerable potential to act as an aid in diagnosis at acute time points post-TBI as evidenced by the ability to discriminate between TBI and controls; AUCs ranged from 0.68 to 0.94 with the majority of AUCs exceeding 0.80^22,28–38^ (Table 2). However, GFAP’s ability to discriminate TBI from controls varied by context of use and control group with the highest AUCs reported in ED studies using uninjured patients as controls (0.85–0.96),^28,30,31,37,38^ followed by ED studies using ED trauma patients as controls (0.68–0.92),^22,29,31,35,37^ and lowest in studies of athlete cohorts (0.57–0.86).^34,36,40,41^ GFAPs more modest discriminatory ability when injured controls are used might reflect the effect of occult brain injury in nonconcussed patients with ED trauma^45^ and nonconcussed contact athletes^46^ serving to obscure between-group GFAP differences. Combining GFAP with UCH-L1 levels increased GFAP’s diagnostic AUCs by only 0.04–0.05.^30,35,40,42^

**Table 2.** Diagnostic Utility (Traumatic Brain Injury vs. No Traumatic Brain Injury) of Glial Fibrillary Acidic Protein, Ubiquitin C-Terminal Hydrolase L1, and S100B at Acute Time Points

Time postinjury (h)	Patient with TBI group	Comparison control group	AUC (95% CI)	Reference
GFAP and UCH-L1				
0–4	Patients with ED with GCS 9–15 (90% GCS 13–15)	Uninjured controls ED trauma controls	0.90 (0.86–0.94) GFAP	Papa (2012)^31^
			0.68 (0.56–0.78) GFAP	
0–4	Patients with ED with GCS 9–15 (90% GCS 13–15)	Uninjured controls ED trauma controls	0.87 (0.82–0.92) UCH-L1	Papa (2012)^39^
			0.67 (0.56–0.78) UCH-L1	
0–24	Patients with ED with GCS 3–15 (83% GCS 13–15)	Uninjured controls	0.91 (0.88–0.94) GFAP	Diaz-Arrastia (2014)^30^
			0.87 (0.83–0.90) UCH-L1	
			0.94 (0.92–0.96) GFAP+UCH-L1	
0–4	Patients with ED with GCS 13–15	ED trauma controls	0.75 (0.70–0.80) GFAP	Papa (2014)^32^
4, 8, 12, 16, 20, 24	Patients with ED with GCS 9–15 (98% GCS 13–15)	ED trauma controls	0.73 (0.68–0.77)–0.83 (0.74–0.91) GFAP	Papa (2016)^22^
			0.63 (0.58–0.68)–0.67 (0.53–0.81) UCH-L1	
0–4	Patients with ED with GCS 13–15	ED head trauma controls vs. CT+ mTBI ED head trauma controls vs. CT- mTBI	0.70 (0.64–0.77) GFAP	Lewis (2017)^33^
			0.65 (0.58–0.74) UCH-L1	
			0.66 (0.59–0.74) GFAP	
			0.60 (0.53–0.71) UCH-L1	
0–24	Hospitalized patients with GCS 13–15 and +CT	Uninjured controls	0.94 (*p* < 0.0001) (95% CI not provided) GFAP	Bogoslovsky (2017)^28^
0–24	Concussed collegiate athletes	Contact sport athletes	0.67 (0.57–0.78) GFAP	Asken (2018)^34^
			0.61 (0.50–0.72) UCH-L1	
0–4	Patients with ED with GCS 15	ED body trauma controls ED head trauma controls	0.76 (0.71–0.80) GFAP	Papa (2019)^35^
			0.69 (0.64–0.74) UCH-L1	
			0.80 (0.73–0.87) GFAP+UCH-L1	
			0.64 (0.54–0.73) GFAP	
			0.54 (0.43–0.66) UCH-L1	
			0.63 (0.53–0.72) GFAP+UCH-L1	
0–1	Concussed high school athletes (Rugby)	Contact sport athletes	0.86 (0.73–0.99) GFAP	Laverse (2020)^36^
0–6	Concussed collegiate athletes	Contact sport athletes	0.68 (0.61–0.75) GFAP	McCrea (2020)^40^
			0.66 (0.58–0.74) UCH-L1	
			0.72 (0.65–0.79) GFAP+UCH-L1	
0–6	Concussed high school athletes	Noncontact sport athletes Contact sport athletes	0.79 (0.70–0.88) UCH-L1	Meier (2020)^41^
			0.63 (0.52–0.74) GFAP	
			0.74 (0.65–0.83) UCH-L1	
			0.57 (0.47–0.66) GFAP	
0–6	Concussed military cadets during combat training	Military cadets	0.75 (0.64–0.87) GFAP	Giza (2021)^42^
			0.73 (0.59–0.86) UCH-L1	
			0.78 (0.66–0.91) GFAP+UCH-L1	
0–24	Patients with ED with GCS 13–15	ED trauma controls	0.92 (0.88–0.97) GFAP	Clarke (2021)^29^
0–6	Patients with ED with GCS 13–15	Uninjured controls	0.85 (0.78–0.93) GFAP	Reyes (2023)^38^
			0.79 (0.70–0.88) UCH-L1	
0–24	Patients with ED with GCS 13–15	Uninjured controls ED trauma controls	0.95 (0.94–0.97)–0.96 (0.94–0.99) GFAP	Gardner (2023)^37^
			0.80 (0.72–0.89)–0.89 (0.86–0.91) UCH-L1	
			0.90 (0.88–0.92)–0.95 (0.91–0.99) GFAP	
			0.62 (0.47–0.77)–0.69 (0.64–0.74) UCH-L1	
S100B				
0–6	Patients with ED with GCS 13–15	Uninjured controls	0.71 (0.68–0.74)	Bazarian (2013)^43^
0–1	Concussed elite athletes	Contact sportathletes	0.67 (0.52–0.83)	Shahim (2014)^44^
0–4	Patients with ED with GCS 9–15	ED trauma controls	0.68 (0.63–0.74)	Papa (2014)^32^
0–4	Patients with ED with GCS 13–15	ED trauma controls	0.69 (0.61–0.77)	Lewis (2017)^33^
0–4	Concussed collegiate athletes	Contact sport athletes	0.75 (0.65–0.85)	Asken (2018)^34^
0–6	Concussed high school athletes	Noncontact sport athletes Contact sport athletes	0.79 (0.70–0.88)	Meier (2020)^41^
			0.68 (0.60–0.77)	

AUC, area under the curve; CI, confidence interval; ED, emergency department; GCS, glasgow coma scale; GFAP, glial fibrillary acidic protein; TBI, traumatic brain injury; UCH-L1, ubiquitin C-terminal hydrolase L1.

#### Prognostic capabilities

GFAP serum/plasma levels have potential to aid in the prediction of mortality and long-term global functional outcome as reflected in its association with Glasgow Outcome Scale (GOS) and GOS–Extended (GOS-E) scores 6–12 months after injury. The GOS classifies outcome into five categories (1 = death, 2 = vegetative state, 3 = severe disability, 4 = moderate disability, and 5 = good recovery), whereas the GOS-E uses an 8-point scale (1 = death, 2 = vegetative state, 3 = severe disability, lower, 4 = severe disability, upper, 5 = moderate disability, lower, 6 = moderate disability, upper, 7 = good recovery, lower, and 8 = good recovery, upper).^47^ Scores from both scales are often dichotomized into favorable versus unfavorable outcome, and complete versus incomplete recovery. AUCs for acute GFAP predicting 6–12 months outcome varied considerably (Table 3). Acute serum/plasma levels of GFAP were better at predicting 6–12 months outcomes (as indicated by larger AUCs) among those with more severe TBIs (AUCs: 0.68–0.82)^51,52^ compared with patients with GCS 13–15 (AUCs: 0.53–0.72).^50,51^ Moreover, acute levels of GFAP were better predictors of long-term outcomes involving mortality (AUCs: 0.72–0.87)^48,51^ and unfavorable outcome (AUCs: 0.67–0.86),^30,48–52^ than of incomplete recovery (0.53–0.68).^30,49–51^ The prediction of mortality and unfavorable outcome was enhanced by combining GFAP results with acute levels of UCH-L1, as indicated by increases in AUC of 0.03–0.09.^30,51^ GFAP serum/plasma levels also contributed incremental value toward prediction of 6 months outcome over and above clinical variables.^53^ When GFAP was added to prognostic models using demographic, clinical, and radiological variables, AUCs predicting outcome increased by 0.01–0.02, corresponding to a 2.8–4.4% increase in model *R*^2^ (Supplementary Table S1). Combining GFAP with UCH-L1 improved GFAPs incremental value to prognostic AUCs by 0.007–0.016, contributing to increases in model *R*^2^ of 3.1–6.4%.

**Table 3.** Prognostic Utility of Glial Fibrillary Acidic Protein, Ubiquitin C-Terminal Hydrolase L1, and S100B at Acute Time Points

Time postinjury (h)	Patient with TBI group	Outcome predicted	AUC (95% CI)	Reference
GFAP and UCH-L1				
0–24	Patients with ED GCS 3–15	Incomplete recovery (GOS-E <8) at 3 months	**0.65** (0.55–0.74) GFAP	Diaz-Arrastia (2014)^30^
			**0.58** (0.50–0.74) UCH-L1	
			**0.64** (0.55–0.72) GFAP+UCH-L1	
		Incomplete recovery (GOS-E <8) at 6 months	**0.60** (0.43–0.77) GFAP	
			**0.51** (0.39–0.63) UCH-L1	
			**0.61** (0.48–0.75) GFAP+UCH-L1	
		Unfavorable outcome (GOS-E <4) at 3 months	**0.74** (0.61–0.87) GFAP	
			**0.80** (0.70–0.90) UCH-L1	
			**0.83** (0.75–0.91) GFAP+UCH-L1	
		Unfavorable outcome (GOS-E <4) at 6 months	**0.74** (0.61–0.87) GFAP	
			**0.76** (0.60–0.91) UCH-L1	
			**0.81** (0.70–0.91) GFAP+UCH-L1	
Hospital admission	GCS <8	Mortality at 6 months	**0.76** (0.61–0.92) GFAP	Lei (2015)^48^
		Unfavorable outcome (GOS 1–3) at 6 months	**0.82** (0.70–0.94) GFAP	
Hospital admission	GCS 3–15	Unfavorable outcome (GOS 1–3) at 6–12 months	**0.72** (0.60–0.81) GFAP	Takala (2016)^49^
			**0.73** (0.61–0.82) UCH-L1	
		Incomplete recovery (GOS-E <8) at 6–12 months	**0.63** (0.52–0.71) GFAP	
			**0.54** (0.43–0.63) UCH-L1	
0–24	Patients with ED GCS 13–15	Unfavorable outcome (GOS-E <4) at 6–12 months Incomplete recovery (GOS-E <8) at 6–12 months	**0.74** (0.63–0.88) GFAP	Hossain (2019)^50^
			**0.60** (0.50–0.71) GFAP	
0–24	Patients with ED GCS 3–12	Mortality at 6 months	**0.79** (0.73–0.85) GFAP	Korley (2022)^51^
			**0.81** (76–0.86) UCH-L1	
			**0.84** (0.80–0.89) GFAP+UCH-1	
		Unfavorable outcome (GOS-E <4) at 6 months	**0.77** (0.73–0.83) GFAP	
			**0.76** (0.71–0.81) UCH-L1	
			**0.81** (0.77–0.86) GFAP+UCH-1	
		Incomplete recovery (GOS-E <8) at 6 months	**0.68** (0.61–0.76) GFAP	
			**0.65** (0.56–0.74) UCH-L1	
			**0.70** (0.63–0.76) GFAP+UCH-L1	
0–24	Patients with ED GCS 13–15	Mortality at 6 months	**0.72** (0.60–0.84) GFAP	Korley (2022)^51^
			**0.77** (0.66–0.88) UCH-L1	
			**0.78** (0.66–0.89) GFAP+UCH-1	
		Unfavorable outcome (GOS-E <4) at 6 months	**0.67** (0.58–0.76) GFAP	
			**0.70** (0.62–0.79) UCH-L1	
			**0.72** (0.64–0.80) GFAP+UCH-1	
		Incomplete recovery (GOS-E <8) at 6 months	**0.53** (0.50–0.56) GFAP	
			**0.53** (0.50–0.56) UCH-L1	
			**0.53** (0.50–0.56) GFAP+UCH-L1	
0–24	Patients with ED GCS 3–15	Mortality at 6 months	**0.87** (0.83–0.91) GFAP	Korley (2022)^51^
			**0.89** (0.66–0.88) UCH-L1	
			**0.90** (0.87–0.94) GFAP+UCH-1	
		Unfavorable outcome (GOS-E <4) at 6 months	**0.86** (0.83–0.89) GFAP	
			**0.86** (0.86–0.92) UCH-L1	
			**0.89** (0.86–0.91) GFAP+UCH-1	
		Incomplete recovery (GOS-E <8) at 6 months	**0.62** (0.59–0.64) GFAP	
			**0.61** (0.59–0.64) UCH-L1	
			**0.62** (0.60–0.65) GFAP+UCH-L1	
0–24	Patients with ED with GCS 3–15	Unfavorable outcome (GOS-E <4) at 6 months	**0.78** (0.65–0.92) GFAP	Castaño-Leon (2022)^52^
			**0.77** (0.65–0.89) UCH-L1	
S100B				
See text				

AUC, area under the curve; CI, confidence interval; ED, emergency department; GCS, glasgow coma scale; GFAP, glial fibrillary acidic protein; GOS-E, glasgow outcome scale–extended; TBI, traumatic brain injury; UCH-L1, ubiquitin C-terminal hydrolase L1.

#### Limitations

Normative GFAP levels differ by age groups and are elevated in those <10 and >59 years of age, pointing out the need for age-specific cutoffs.^54^ Acute elevations in serum/plasma GFAP levels are also seen in neurological conditions such as Alzheimer’s disease, multiple sclerosis, and acute ischemic stroke,^55–58^ although levels are usually much lower than levels seen after more severe TBI. These well-documented elevations of serum GFAP in other acute and chronic neurological diseases underscore the nuanced interpretation of clinical context required when assessing GFAP levels in the evaluation of TBI. In addition, cutoffs related to the risk of CT-detected ICI vary by analysis platform and manufacturer, underscoring the need for certified reference materials that would allow cross-calibration across assay platforms. Moreover, it is unclear the extent to which the addition of serum/plasma levels of UCH-L1 to GFAP levels improves the prediction of CT-detected ICI over GFAP levels alone. Finally, there are no validated GFAP cutoffs identifying those at very high risk of ICI, which has particular relevance in military operations and other austere settings where access to CT scanning is limited.

**UCH-L1:** UCH-L1 is a deubiquitinating enzyme that helps break down damaged or excess proteins in cells, facilitating their disposal.^59^ UCH-L1 has also been linked to proteinopathy-linked neurodegenerative diseases (e.g., Parkinsonism, and Alzheimer’s disease).^59^ UCH-L1 is highly expressed in neurons, detectable in the blood within 30 min of TBI, peaks at 8 h postinjury, declines rapidly over 48 h, and has a serum/plasma half-life of 7–9 h.^21,22,60^

#### Prediction of traumatic ICI on CT scan

Like GFAP, UCH-L1 measured acutely postinjury in serum/plasma can assist in identifying head-injured patients who are at very low risk for ICI on head CT scan. As with GFAP, the UCH-L1 cutoffs defining high sensitivity and NPV were determined in studies in which it was combined with GFAP serum/plasma levels. At UCH-L1 cutoffs of 327–400 pg/mL (depending on the assay), tests combining acute measurements of UCH-L1 with GFAP had sensitivities ranging from 0.957 to 1.00 and NPVs from 0.965 to 1.00^8,11–15^ (Table 1). However, specificities and PPVs were notably lower with AUCs ranging from 0.36 to 0.41 and from 0.050 to 0.10, respectively. In combination with GFAP, UCH-L1 has been FDA- and EMA-cleared for predicting normal CT scans, and has been endorsed in the French Mild TBI Practice Guidelines.^9^

#### Aid in diagnosis

Serum/plasma levels of UCH-L1 can potentially aid in diagnosing TBI acutely by discriminating between patients with TBI and control patients without TBI. However, the accuracy of this discrimination depends on the context of use and the nature of the control group (Table 2). AUCs were the highest in ED studies using uninjured patients as controls (0.79–0.89),^30,37–39^ followed by studies using contact athlete controls (0.61–0.79),^34,40,41^ and lowest in ED studies using ED trauma patients as controls (0.62–0.69).^22,35,37,39^ Again, this pattern might be explained by occult brain injury in nonconcussed ED trauma patients^45^ and nonconcussed contact athletes^46^ obscuring between-group GFAP differences.

#### Prognostic capabilities

Serum/plasma UCH-L1 measured within 24 h of injury is able to predict mortality and global functional outcomes 6–12 months postinjury. Like GFAP, UCH-L1’s prognostic ability varied depending on the outcome examined and the initial TBI severity (Table 3). Acute serum/plasma levels of UCH-L1 were better able to predict 6- to 12-month outcomes among those with more severe TBIs (AUCs: 0.65–0.81)^51^ compared to patients with GCS 13–15 (AUCs: 0.53–0.77),^51^ although this is based on limited data. Similar to GFAP, acute levels of UCH-L1 were better predictors of long-term outcomes involving mortality (AUCs: 0.77–0.89)^51^ and unfavorable outcome (AUCs: 0.73–0.86),^30,49,51,52^ compared to predicting incomplete recovery (AUCs: 0.51–0.65).^30,49,51^ Combining acute UCH-L1 results with those from GFAP improved UCH-L1’s prediction of 6–12 months unfavorable outcome and incomplete recovery (AUCs increased 0.05–0.10), but this effect was limited to cohorts containing patients with GCS <13.^30,51^ When UCH-L1 serum/plasma was added to prognostic models using demographic, clinical, and radiological variables, AUCs predicting outcome increased by 0.02–0.03, corresponding to a 3.7–6.6% increase in model *R*^2^ values^53^ (Supplementary Table S1).

#### Limitations

UCH-L1’s relatively short half-life in blood may reduce its diagnostic and prognostic utility at acute time points beyond 12 h. In addition, reference ranges have not been established, there are no certified reference methods and materials for assay standardization, and cutoffs related to the risk of CT-detected ICI vary by analysis platform and manufacturer. Finally, the observation that UCH-L1 serum/plasma concentrations in CT-negative patients with TBI are significantly higher in males than females underscores the need for sex-specific cutoffs.^61^

It remains an open question whether UCH-L1 measurements add value over GFAP measurements alone.^62^ Since UCH-L1 peaks earlier than GFAP,^22^ it is possible that UCH-L1 levels are needed in the very early postinjury time period. However, most prior studies contain few samples collected within the first hour of injury.^8,51,53^ A 2024 study on GFAP and UCH-L1 levels in samples collected within 30 min of injury found no added benefit of UCH-L1 in subjects with GCS scores <13,^21^ leaving open the possibility that UCH-L1 could add discriminant value at very early time points in those with GCS 13–15.

**S100B:** S100B is a calcium-binding protein that exists as S100 A1B and S100 BB dimers^63^ and is concentrated primarily by astrocytes (but also chondrocytes, melanocytes, and skeletal muscle cells) and functions to regulate cellular calcium homeostasis, promote neurite extension, and inhibit protein kinase C-mediated phosphorylation.^64^ S100B has a short half-life of 0.5–2 h when released into the bloodstream after TBI.^65–67^

#### Prediction of traumatic ICI on CT scan

Similar to GFAP and UCHL1, S100B serum/plasma can assist in identifying individuals who are likely to have a normal head CT scan, potentially avoiding the necessity for immediate neuroimaging. However, the sampling time frame of 3–6 h postinjury is shorter than other biomarker proteins because of S100B’s short half-life. At a cutoff of 0.105 μg/L measured in serum/plasma within 3 h of injury in those with GCS 13–15, S100B has a sensitivity of 0.988 and NPV of 0.997 (Roche product insert). Specificity and PPV were 0.329 and 0.11, respectively (Table 1). Validated as part of Scandinavian TBI clinical decision-making guidelines using a cutoff of 0.10 μg/L among low-risk mild patients with TBI within 6 h of injury, S100B had slightly lower sensitivity (0.94) and specificity (0.19) in this demographic cohort.^67,68^ Additionally, S100B is recommended for use in the French TBI clinical decision guidelines.^9^ S100B demonstrates similar performance among pediatric cohorts, with sensitivities ranging from 97% to 100% and specificities ranging from 34% to 37.5%.^69^

#### Aid in diagnosis

Multiple studies have demonstrated elevations in S100B serum/plasma after TBIs in ED settings and athletic cohorts.^70^ However, to date, S100B has only a moderate ability to discriminate TBI from controls with AUCs ranging from 0.67 to 0.79^32–34,41,43,44^ (Table 2).

#### Prognostic capabilities

The prognostic utility of S100B measured at acute time points has been demonstrated primarily in those with GCS <13. A sensitivity analysis of 12 studies (770 patients with TBI) in a 2013 systematic review estimated the mean S100B levels measured within 24 h of TBI were 2.6 times higher (95% CI: 2.0–3.2) in patients who died in hospital compared to those who survived.^71^ A sensitivity analysis of 18 studies (933 patients with TBI) in the same review estimated the mean S100B concentrations among those with unfavorable outcome (GOS ≤3) at 3, 6, or 12 months to be 2.6 times higher (95% CI: 2.1–3.4) than those with a favorable outcome. In a separate study, peak S100B during the first day postinjury had a respectable ability to predict unfavorable outcome at 6 and 12 months with AUCs of 0.81 and 0.83, respectively^72^ (Table 4). S100B was also found to contribute to incremental value toward the prediction of 6-month postinjury outcome over and above demographic, clinical, and radiological variables (Supplementary Table S1).^53^

**Table 4.** Prognostic Utility of Neurofilament Light Chain, Glial Fibrillary Acidic Protein, and S100B at Subacute Time Points

Time postinjury	Patient with TBI group	Outcome predicted	AUC (95% CI)	Reference
NfL				
1–24 h	GCS 13–15	Unfavorable GOS-E (≤4) at 6–12 months	**0.83** (0.69–0.96)	Hossain (2019)^50^
24 h	GCS <8	Unfavorable GOS (1–3) at 12 months	**0.71** (95% CI not provided)	Shahim (2016)^73^
6 days	Concussed elite athletes	Post-concussive symptoms at 12 months	**0.97** (0.94–1.0)	Shahim (2020)^74^
10 d–6 wk [peak concentration]	GCS 3–15	Unfavorable GOS-E (≤5) at 6 months Unfavorable GOS-E (≤5) at 12 months	**0.83** (0.75–0.91)	Graham (2021)^72^
			**0.85** (0.77–0.94)	
GFAP				
1–5 days	GCS <8	Mortality at 6 months	Ranging from **0.79** to **0.77** (95% CI not provided)	Lei (2015)^48^
		Unfavorable GOS (1–3) at 6 months	Ranging from **0.69** to **0.85** (95% CI not provided)	
7 days	GCS 4–8	Unfavorable GOS (1–3) at 6 months Unfavorable GOS (1–3) at 12 months	**0.76** (0.61–0.90)	Raheja (2016)^75^
			**0.82** (0.65–0.98)	
		Mortality at 6 months Mortality at 12 months	**0.88** (0.71–1.00)	
			**0.81** (0.61–1.00)	
Day 1 [peak concentration]	GCS 3–15	Unfavorable GOS-E (≤5) at 6 months Unfavorable GOS-E (≤5) at 12 months	**0.73** (0.63–0.82)	Graham (2021)^72^
			**0.78** (0.69–0.86)	
S100B				
Day 1 [peak concentration]	GCS 3–15	Unfavorable GOS-E (≤5) at 6 months Unfavorable GOS-E (≤5) at 12 months	**0.81** (0.72–0.89)	Graham (2021)^72^
			**0.83** (0.74–0.91)	

AUC, area under the curve; CI, confidence interval; ED, emergency department; GCS, glasgow coma scale; GFAP, glial fibrillary acidic protein; GOS, glasgow outcome scale; GOS-E, glasgow outcome scale–extended; NfL, neurofilament light chain.

#### Limitations

S100B increases with age, necessitating age-specific cutoffs,^76^ although population-based reference ranges in younger age groups have been established.^77,78^ S100B has not been standardized across assays and platforms; therefore, reference limits and cutoff points need to be interpreted in relation to laboratory-specific data. S100B elevations in other conditions such as melanoma,^79^ trauma to tissues outside the brain (bone fractures, severe soft tissue injuries),^80^ intense physical exertion,^81^ epilepsy,^82^ and stroke^83^ have the potential to reduce specificity in certain patients. Few studies have compared S100B with GFAP head-to-head. In a population of mostly mild patients with TBI, serum/plasma collected within 4 h of injury GFAP significantly outperformed S100B with AUC for predicting CT lesions for GFAP of 0.84 (0.73–0.95) and for S100B 0.78 (0.67–0.89).^32^ In patients with TRACK-TBI with more severe TBI with serum/plasma collected later within 24 h of injury GFAP significantly outperformed S100B for discriminating between CT-positive from CT-negative TBI cases AUC 0.85 (95% CI: 0.83–0.87) for GFAP, compared with AUC 0.67 (0.64–0.70) for S100B.^26^ Similar findings were reported by the CENTER-TBI investigators.^20^ One limitation of those studies was that most samples were collected relatively late after injury, and S100B may perform better for samples collected within the first 3–6 h. Finally, as with GFAP and UCH-L1, current S100B cutoffs for prediction of CT-detected traumatic ICI are method- and/or laboratory-specific, indicating the necessity for certified and standardized reliable reference materials and methods.

## Biomarkers with Utility at Subacute Time Points (1–30 Days)

Subacutely measured blood levels of neurofilament light chain (NfL), GFAP, and S100B have good potential to contribute to an improved TBI classification system because of their demonstrated ability to predict neuroimaging abnormalities and poor functional outcome at 6–12 months, primarily among patients with TBI hospitalized in ICU settings. In addition, NfL and GFAP serum/plasma levels can contribute to diagnosis by accurately classifying those with and without TBI at subacute time points.

### Neurofilament light chain

NfL is a component of neurofilaments, which are tube-shaped proteins found only in the cytoplasm of neurons.^84^ Neurofilaments give neurons structural support and are found in dendrites, the cell body, and especially in axons, where they are most common. Because neurofilaments help axons grow in thickness, larger myelinated axons have high levels of neurofilaments and NfL.^85^ NfL is elevated in blood on the first day after TBI, but in distinction to GFAP, UCH-L1, and S100B, levels continue to increase over the first 2–3 weeks after injury. Peak levels are typically over 20-fold higher than on the first day and remain elevated for many months. NfL assays certified by the Clinical Laboratory Standards Institute (CLSI) are available through LabCorp and Mayo Clinic.

#### Aid in diagnosis

NfL serum/plasma levels at 2 weeks and 3 months discriminated patients with GCS 13–15 from trauma controls with an AUC of 0.83 (95% CI: 0.75–0.91) and AUC 0.78 (0.70–0.87), respectively.^29^ NfL levels at 30 days postinjury discriminated healthy controls from patients with TBI with GCS scores of 13–15, 9–12, and 3–8 with AUCs of 0.84, 0.92, and 0.92, respectively (95% CI not provided).^86^

#### Prediction of neuroimaging abnormalities

Blood levels of NfL measured at subacute time points postinjury were found to be associated with white matter atrophy and reduced white matter integrity weeks to months after injury, and correlated with decreases in gray and white matter volumes and lesion volumes on MRI.^72,74^ In a separate study, NfL levels measured at 1–24 h postinjury correlated with reductions in white matter integrity on DTI 4–15 months postinjury.^87^

#### Prognostic capabilities

NfL levels measured within 30 days of injury have the potential to assist in predicting global functional outcomes and persistent symptoms 6–12 months after injury with AUCs ranging from 0.71 to 0.85^50,72–74,86^ (Table 4). NfL measured 30 days after injury in a clinic-based cohort of patients with TBI GCS 3–15 (GCS: 13–15 in 55%) correlated with an improvement in GOS-E at 90 days (ρ = 0.85, *p* = 0.0004).^86^

#### Limitations

NfL has not yet been cleared for use by FDA or other regulatory bodies, although it has received breakthrough device designation from FDA (for use in the evaluation of multiple sclerosis).^88^ Other limitations include the need to develop reference methods and materials for assay standardization and the need for age-specific cutoffs due to elevations among healthy older adults.^54,89^ In addition, NfL, like other BBMs, may not be entirely specific for TBI, as blood level elevations are found in other neurological disorders such as stroke,^58^ amyotrophic lateral sclerosis, multiple sclerosis, and Alzheimer’s disease,^90^ although usually to much lower levels than what is reported within a few weeks after TBI.

### Glial fibrillary acidic protein

#### Aid in diagnosis

At subacute time points postinjury, limited data indicate that GFAP serum/plasma has a reasonable ability to discriminate hospitalized patients with TBI from trauma and healthy controls with AUCs ranging from 0.81 to 0.94^22,86^ (Table 5). Among patients with ED 0–3 days after injury, discrimination was less robust, with a single study reporting an AUC of 0.74.^28^

**Table 5.** Diagnostic Utility (Traumatic Brain Injury vs. No Traumatic Brain Injury) of Neurofilament Light Chain, Glial Fibrillary Acidic Protein, and S100B at Subacute Time Points

Time postinjury	Patient with TBI group	Comparison control group	AUC (95% CI)	Reference
NfL				
1–2 days	Concussed military cadets during combat training	Military cadets	0.66 (0.54–0.78)	Giza (2021)^42^
GFAP				
1–7 days	Hospitalized patients with GCS 9–15	Trauma controls	Ranging from **0.81** (0.70–0.93) to **0.94** (0.78–1.00)	Papa (2016)^22^
30 days	Hospitalized patients with GCS 9–12 Hospitalized patients with GCS 3–8	Healthy controls	**0.89** (95% CI not provided) **0.89** (95% CI not provided)	Shahim (2020)^86^
0–3 days	Patients with ED with GCS 13–15	Trauma controls	**0.74** (0.69–0.80)	Clarke (2021)^29^
1–2 days	Concussed military cadets during combat training	Military cadets	**0.63** (0.51–0.75)	Giza (2021)^42^
S100B				
See text				

AUC, area under the curve; CI, confidence interval; ED, emergency department; GCS, glasgow coma scale; GFAP, glial fibrillary acidic protein; GOS, glasgow outcome scale; NfL, neurofilament light chain.

#### Prognostic capabilities

Several studies suggest that subacutely measured serum/plasma GFAP has moderate to good predictive capability with AUCs for 6- to 12-month mortality and unfavorable functional outcomes ranging from 0.69 to 0.88^48,75^ (Table 4). GFAP levels measured 30 days after injury in a clinic-based cohort of mixed-severity patients with TBI correlated with improvements in GOS-E scores at 90 days.^74^

#### Prediction of neuroimaging abnormalities

Limited evidence suggests that increases in GFAP between 1 and 7 days postinjury were quite good at distinguishing those with and without traumatic ICI on head CT scan, with AUCs ranging from 0.82 to 0.97.^22^ These AUCs did not appreciably increase with the addition of UCH-L1. Moreover, peak GFAP serum/plasma levels during the first 6 weeks postinjury among patients with GCS scores of 3–12 correlated with lesion volume on MRI.^6^

#### Limitations

The limitations of GFAP blood levels at subacute time points postinjury are similar to their limitations at acute time points and include the lack of certified reference methods and materials for assay standardization, as well as potential reductions in specificity due to elevations in other conditions.

### S100B

#### Diagnostic capability

Although several studies have reported post-TBI elevations of serum/plasma S100B at subacute time points,^91,92^ to our knowledge none have estimated its ability to distinguish between patients with and without TBI beyond the acute phase of injury.

#### Prognostic capabilities

S100B has a reasonable ability to predict mortality, as evidenced by a meta-analysis of nine studies indicating that levels within 24 h in hospitalized patients predict in-hospital mortality and mortality within 1 year of injury, with average AUCs of 0.83 ± 0.13 and 0.73 ± 0.09, respectively.^93^ Peak S100B levels during the initial 6 weeks postinjury among those with GCS scores of 3–12 (usually occurring within the first day of injury) were found to predict 6- and 12-month unfavorable outcome with an AUC of 0.81 and 0.83, respectively.^72^ Furthermore, several studies evaluating those with GCS <13 suggest that S100B levels measured between 12 and 36 h have stronger predictive power than levels measured at earlier or later time points.^94,95^

#### Prediction of neuroimaging abnormalities

Limited data suggest that subacute S100B serum/plasma levels can herald the development of delayed pathology on neuroimaging. Individual-specific increases in S100B levels 48 h or more postinjury in hospitalized patients with TBI anticipated, by a mean of 2 days, the emergence of new pathology visible on CT or MRI, with an AUC of 0.855.^96^ Furthermore, an S100B increase of >0.05 μg/L had 80% sensitivity and 89% specificity for predicting new neuroimaging pathology.^96^ In a separate study, peak S100B levels during the initial 6 weeks postinjury among those with GCS scores of 3–12 (usually occurring within the first day of injury) correlated with lesion volume on MRI acquired at 1–6 weeks and with unfavorable GOS at 6 and 12 months^72^ (Table 4).

#### Limitations

The limitations of S100B levels at subacute time points are similar to their limitations at acute time points including confounding by release from injuries outside the brain such as fractures, major lacerations, and crush injuries. Another limitation is the lack of assay standardization.

## Biomarkers with Utility at Chronic Time Points (**>**30 Days)

The working group was unable to identify substantial data supporting the utility of BBMs measured more than one month after injury for contributing to a revised TBI classification system. However, three markers, NfL, GFAP, and phosphorylated tau (p-Tau), showed promise (see Supplementary Data). These three BBMs have also been widely studied as indicators of neurodegenerative pathology, including Alzheimer’s disease, frontotemporal dementia, and motor neuron disease.^97^

## Recommendations for Improved System for Characterization and Classification

Current evidence supports the incorporation of serum/plasma levels of GFAP, UCH-L1, and S100B into the classification of TBI at acute time points (0–24 h) postinjury, while NfL, GFAP, and S100B have utility at subacute time points (1–30 days). Blood levels of these biomarkers reflect the extent of structural brain injury in TBI and may be useful for describing the extent of structural brain injury in a classification system. While there is insufficient evidence to support a role for these BBMs at chronic time points (>30 days) postinjury, emerging evidence suggests that NfL, GFAP, and p-Tau isoforms may have a potential future role in this regard.

## Clinical Applications

Historically, the absence of validated biomarkers in neurotrauma has hindered our understanding of TBI and impeded drug development. The recent regulatory approval of GFAP and UCH-L1 as BBMs has the potential to transform clinical evaluation and management of TBI across various contexts. Most TBI biomarker research has focused on ED settings, where studies show that routine testing of GFAP and UCH-L1 could reduce cranial CT usage by about one-third if widely implemented.^8^ However, clinical implementation will depend on demonstrating the added value of BBMs to current clinical criteria for selecting patients for head CT scanning. Validated clinical decision rules, such as the Canadian CT Head Rule (CCHR), the New Orleans Criteria (NOC), and the National Emergency X-Radiography Utilization Study II (NEXUS II), have provided clinical criteria for selective use of head CT scanning among patients with ED with a GCS score above 12.^98–100^ The sensitivity of these rules for detecting traumatic ICI is quite high, with less than 2% false negatives. However, the impact of CDRs on reducing unnecessary head CT scans has been only modest (5% reduction),^101^ with some suggesting they may actually increase CT use.^102,103^ This is reflected in emergency physician confidence in CDRs, with only 56–61% feeling very comfortable or comfortable using these rules and a higher proportion (86%) indicating they would consider a blood test useful.^104^

A 2022 study comparing the performance of GFAP/UCH-L1 to CCHR, NOC, and NEXUS II (completed by clinicians at the time of assessment) found that the combination of the CCHR and GFAP (with or without UCH-L1) provided the highest AUC (0.88, 95% CI: 0.81–0.95) and sensitivity/specificity for predicting traumatic injury on head CT^104^ (Supplementary Table S2). Similar findings were reported in a 2020 study in which GFAP outperformed clinical decision rules (AUC: 0.85, 95% CI: 0.83–0.87 vs. 0.71–0.74 for rule-based models), while combining GFAP with rule components slightly improved discrimination (AUC: 0.86–0.87, 95% CI: 0.85–0.88).^20^

In addition to predicting traumatic abnormalities on head CT scan, routine use of BBMs has the potential to enhance diagnostic accuracy of TBI in acute settings. Several studies report that up to 50% of patients presenting to EDs meeting clinical criteria for mild TBI do not get a TBI diagnosis upon discharge.^105–108^ The impact of these missed diagnoses is potentially substantial. A systematic review of “Diagnostic Errors in the Emergency Department” prepared for the US Agency for Health Care Research and Quality reported that TBI was among the top 15 conditions causing serious misdiagnosis-related harms reported in ED malpractice claims (9th) between 2006 and 2025, and ranked 6th based on total claims payouts ($27 million).^109^

BBMs also have the potential to facilitate better individualized counseling for patients, families, and caregivers at discharge. Many patients with normal cranial CT scans leave the ED without a TBI diagnosis and miss essential follow-up advice, potentially leading to long-term symptoms.^107,108^ Elevated biomarker levels postinjury, even with normal CT findings, provide objective evidence of TBI, which is crucial for optimizing referrals to rehabilitation services.

Biomarkers hold great potential to transform prehospital care for TBI. Less than half of patients with TBI receive evaluation in EDs, yet those not seeking care still face significant disability risks. Utilizing biomarkers at athletic events, urgent care centers, and outpatient clinics could enhance diagnosis, prognosis, and the efficient use of ED and rehabilitation services. Emergency medical personnel could use biomarker tests at injury scenes to guide transport decisions, especially in resource-limited settings like combat zones, rural areas, or mass casualty events. Rising TBI rates in remote regions due to increased motorization in developing countries^110^ and recreational activities in the global north^111^ highlight the potential of BBM rapid tests, though this remains unexplored.

In-hospital ICUs, BBMs can help identify patients at risk for secondary brain injuries and guide decisions on whether to manage them in a neurological ICU or a general trauma ICU. These biomarkers may indicate the need for invasive intracranial monitoring of pressure and brain tissue hypoxia, which are early signs of secondary injury.^66^ Additionally, BBM results can influence care intensity and assess situations where aggressive treatment might be futile. However, some experts argue that S100B should not be used to determine brain death or to guide decisions about withholding treatment in severe TBI cases.^70^

In military settings, BBMs can significantly aid in decision-making regarding return to duty for those without TBI, triaging casualties with TBI and prioritizing evacuation. With head CT scans often unavailable in combat, injured service members may need to be airlifted to CT-equipped/neurosurgeon-staffed facilities, a process that is both risky and resource-intensive. This need is accentuated by the projected limitation in access to neurosurgeons in future military conflicts. The FDA’s 2021 clearance of point-of-care tests for GFAP and UCH-L1 enables these biomarkers to be used in-theater, enhancing the identification of those requiring urgent evacuation for CT scans. The Department of Defense has integrated GFAP and UCH-L1 testing into its Joint Trauma System Clinical Practice Guideline, CPG ID:90.^112^ However, establishing upper-end cutoffs for BBMs to indicate an increased risk of needing neurosurgery is essential. A recent study found that plasma GFAP levels above 372 pg/mL and UCH-L1 above 1231 pg/mL within 12 h of injury were 57× and 28× more likely, respectively, to be associated with traumatic ICI on head CT than those below.^11^ In another study, patients with TBI with GFAP levels above 6200 pg/mL within 30 min of injury were at the highest risk of clinically important outcomes (such as neurosurgical intervention, mechanical ventilation, and death) within 7 days of injury.^21^ These cutoffs require validation to ensure they effectively predict not just ICI but also the need for urgent neurosurgical intervention.

In the postacute settings, including rehabilitation centers and outpatient clinics, biomarkers may help identify patients at risk of late complications from TBI, such as neurodegeneration and post-traumatic epilepsy. Finally, BBMs have a significant potential use for objective outcome measures in TBI clinical trials. For treatment trials, BBMs can indicate the intervention’s path of success and link to physiological and functional measures. In diagnosis trials, these BBMs may aid in verifying diagnostic and prognostic technology candidates, again linking to physiological clinical endpoints.

Determining the cost-effectiveness of BBMs as a prehead CT screening tool is challenging at this early stage of development and clinical adoption. Estimates of cost-effectiveness for this use vary based on multiple factors that differ by institution, region, and country. These factors include the clinical threshold for ordering a head CT, the proportion of positive CT scans, the likelihood that patients with positive scans will receive different treatments (e.g., surgery, hospitalization, ED observation, or immediate discharge), and the associated costs of those treatments, as well as the costs of BBM testing and CT scanning with radiology interpretation.

Cost analyses of S100B suggest that it is cost-neutral in the Scandinavian health care system^113^ but may increase costs in the United States.^114^ An analysis of GFAP/UCHL1 in France estimated a modest potential mean per-person cost savings of €4.15 ± €26.58.^115^ In the United States, a study concluded that cost savings from CT screening with GFAP/UCHL1 depend on the test’s price and the probability of detecting ICI on CT. For patients with a low probability of ICI (10.4%, typically mild TBI), cost-effectiveness could theoretically be achieved at a test cost below $308.96. In those with a higher probability of ICI (66.3%, typically moderate to severe TBI), cost-effectiveness could be achieved at a test cost below $73.41.^116^ Data from real-world experiences using BBMs in clinical environments will be required to better define the true cost-effectiveness of these biomarkers.

## Future Research Applications

Much research remains to be done for BBMs to fulfill their potential to revolutionize brain injury medicine. GFAP, UCHL1, S100B, NfL, and p-Tau isoforms are only the first biomarkers to be widely studied. Recent research has focused on biomarkers that have promise for measuring pathophysiologic endophenotypes, such as neuroinflammation^117–119^ and traumatic microvascular injury.^120,121^ Biomarkers are sensitive enough to detect changes in function that do not rise to the level of overt, diagnosable injury (i.e., following exposures to low-level blast or repetitive subconcussive head impacts), but that may convey increased risk of injury with subsequent exposures will also be useful for establishing mitigation strategies in military and athletic populations.

## Recommendations for Biomarker Analysis and Interpretation

### Establish appropriate reference ranges for clinical decision-making

BBMs have potential uses in TBI: (1) distinguishing injured from noninjured; (2) indicating injury severity; (3) identifying pathophysiologic endophenotypes (e.g., excitotoxicity, inflammation); and (4) predicting recovery. In each of these contexts, BBMs need reference ranges for accurate interpretation. As brain injury biomarkers become part of clinical practice, refining these ranges across diverse populations—considering factors like age, sex, comorbidities, polypharmacy, and potentially other lifestyle factors—will be essential. BBMs should not function alone but alongside imaging, clinical indicators, and personalized modifiers to provide a clinically meaningful estimate of TBI severity.

#### Individual baseline comparison versus normalized reference ranges

Biomarker measures can be compared using two reference points: (1) a patient’s individual baseline or (2) a normalized reference range. Ideally, each patient would have a personal baseline for comparison, but this isn’t feasible in current clinical settings. For now, the best alternative is to compare biomarkers to normalized reference ranges. However, without standardized reference methods, such ranges should be viewed cautiously, as they can vary across labs. Establishing standardized reference ranges for specific biomarkers in healthy populations is crucial, especially for biomarkers like GFAP, UCH-L1, and NfL. A study in Canada highlighted that some biomarkers are naturally elevated in healthy older adults (>60 years) and prepubescent children.^54^ Reference ranges should be based on data from diverse populations, considering factors like age, sex, and race/ethnicity. To develop accurate ranges, it is essential to recruit at least 120 healthy individuals per demographic group, as recommended by CLSI guidelines.^122^ Stratified sampling by key factors like age and sex is important to ensure reference ranges are relevant for different populations. Standardized assays and laboratory-specific quality control measures are necessary to maintain consistent biomarker measurements over time.

#### Specialized population considerations

BBMs must account for patient-specific factors when setting reference ranges. For instance, high GFAP levels may indicate TBI but could also be elevated in older adults or those with neurodegenerative diseases. Conversely, GFAP may be deceptively low in patients with high body mass index (BMI), diabetes, or tobacco use.^123^ Excluding individuals with comorbidities from research has limited the ability to establish accurate reference ranges necessary for widespread biomarker applicability. Biological variability across different contexts should be studied to improve the interpretation of serial measurements. Ongoing refinement of reference ranges based on factors like age, sex, and comorbidities is essential for accurate biomarker use. Although these biomarkers are brain-enriched, their expression is not exclusively restricted to the brain. As a result, modestly elevated levels are observed in patients with polytrauma, including those with fractures and peripheral injuries. Further research is needed to understand better how nonbrain injuries influence levels of these biomarkers.

### Harmonize/standardize assays across platforms

To ensure blood-based protein biomarkers accurately reflect injury status, standardized thresholds must be established for specific analytes. Cross-assay reliability is essential, requiring consistent results across different laboratory platforms. Achieving this involves harmonizing and standardizing assays, a challenge being addressed in other neurological conditions like Alzheimer’s disease, frontotemporal dementia, and amyotrophic lateral sclerosis.^97^ Key efforts include developing certified reference methods and materials for assay standardization, with ongoing work by the IFCC Biomarkers for Neurodegenerative Diseases working group (WG-BND).^124^

#### Robustness of biomarker assays to variations in preanalytical factors

To ensure biomarker assays are robust for clinical use, additional validation on existing assay platforms is crucial. The effect of preanalytical factors, which account for 50–70% of test errors, on assay performance must be established.^125,126^ These factors include collection-to-processing time, stability as a function of freezer time and freeze–thaw cycles, diurnal and postprandial effects repeatability, and reproducibility across different reagent lots, instruments, and laboratory technicians. In addition, variations in sample collection and processing, such as the fraction of the blood used for testing (plasma, serum, capillary whole blood)^127,128^ and the presence or absence of additives such as Ethylenediaminetetraacetic acid (EDTA), can also substantial sources of variability in laboratory testing.^129^

#### Analytical precision

TBI biomarker assays must undergo rigorous analytical validation to ensure accuracy, precision, selectivity, sensitivity, reproducibility, and stability.^130^ This process confirms the assay’s reliability for clinical and research use. Key validation factors include accuracy (how close results are to true values), precision (consistency of repeated measurements), linearity (response across expected biomarker levels), and selectivity (ability to distinguish the biomarker from other substances). Without thorough validation, biomarker results cannot be trusted or meaningfully interpreted. Numerous peer-reviewed studies provide guidelines on analytical validation,^131–133^ making this process essential for the development of reliable brain injury biomarker assays.

Improve transparency in assay development through large-scale data sharing of methods and results. There is a significant lack of standardization and transparency in assay design and methodology, making it difficult to compare biomarker results across studies. For instance, two research groups using ELISA to quantify GFAP in blood post-TBI could yield entirely different values due to variations in assay design. Key factors like the protein subunit used, antibodies and their epitope targets, washing and blocking techniques, and plate reader settings all influence output signals, affecting whether clinical thresholds are met. Without transparency in these methods, comparing results between labs becomes nearly impossible, even for the same protein target. Researchers have highlighted these issues with consistency and quality control in TBI biomarker methods.^134–136^ To improve comparability, a centralized hub for sharing methodologies, data, and protocols could help researchers align their techniques. Additionally, developing standardized reference reagents, such as a “universal calibrator” library of qualified protein standards and pooled TBI samples, is crucial for assay consistency across platforms. Academia and industry participation are essential, with assay manufacturers encouraged to disclose details about the proteins measured, their intact or degraded forms, and post-translational modifications. Transparency around target epitopes for capturing antibodies would further enhance comparability, advancing biomarker research and its clinical application.

## Limitations

We identified several key limitations for the inclusion of BBMs into a revised TBI classification system. First, there is a relative lack of data establishing population-based reference ranges, particularly in pediatric and older adult populations, and by sex.^54,137,138^ Second, variability in biomarker assay performance, epitopes targeted, and the fluid component analyzed (plasma, serum, whole blood) complicates efforts to establish marker-specific reference standards and to define cutoffs for different contexts of use and underscores the need for assay harmonization. Third, the absence of certified reference methods, materials, and equations for standardizing BBM assays adds to the challenge.^139,140^ Further, research correlating BBMs with TBI pathoanatomic endophenotypes, akin to Alzheimer’s A/T/N system, is limited, as is BBM prognostic value in older adults and pediatric populations, and after repetitive nonconcussive head hits. Additionally, there is a lack of understanding regarding the impact of physiological factors (e.g., fasting, circadian rhythm, sleep) and comorbid conditions (e.g., stroke, neurodegenerative, cerebrovascular disease, epilepsy) on the diagnostic and prognostic utility of BBMs.^141–144^ Other factors likely to influence BBM values, such as preinjury health, brain barrier integrity,^145^ glymphatic system activity,^146^ extracellular matrix proteolysis,^147^ and renal function,^148^ have also received limited attention. Moreover, longitudinal studies are scarce, with most studies reporting values at a single time point after injury, providing only a snapshot of the dynamic pathophysiology.^22,60,86^

## Future Directions

### Improved BBM standards

As previously stated, it will be essential to develop certified reference methods and materials to standardize the measurement of the most promising biomarkers. Furthermore, it is necessary to refine reference ranges for biomarkers such as S100B, GFAP, UCH-L1, and NfL in diverse populations, considering factors such as age, sex, comorbidities, polypharmacy, and unique exposure risks (e.g., military and athletic populations).

### Promote clinical adoption

For BBMs to be embraced as part of a new TBI classification system, the results need to be as easy for clinicians to access as determining the GCS. BBM results should be available within minutes and simple to interpret. This underscores the need for the development and widespread distribution of easy-to-operate, cost-effective, and rapid point-of-care analyzers. This is critical not only to manage head injuries in-hospital settings, but also in prehospital settings as well as combat environments and athletic fields. Efforts to develop methods for making continuous BBM values easy to interpret will be important for ensuring clinician acceptance of BBMs as part of a TBI classification system. Similarly, pragmatic clinical trials could be useful for demonstrating the clinical utility of BBMs outside of the confines of a research protocol, which will be particularly important to clinicians working in resource-strained settings such as EDs, military environments, and prehospital settings.

### Correlate BBMs with advanced neuroimaging

Future work should determine the relationship of established BBM cutoffs with advanced neuroimaging metrics of probable TBI pathology to confirm, for example, whether measurable evidence of TBI may still be observed in patients within normal ranges. Correlation with advanced neuroimaging can also be used to identify pathoanatomic endophenotypes, such as axonal injury, microglial activation, and oxidative stress, potentially opening the door to use BBMs to monitor the effectiveness of therapeutic interventions targeting these endophenotypes.

### Improve the utility of existing BBMs

Research emphasis should be placed on determining the utility of existing BBMs in older adults and pediatric populations, as well as in individuals with comorbid neurological conditions. In addition, research efforts to determine the BBM cutoffs identifying those at very high risk of traumatic ICI, and perhaps in need of immediate surgery, are likely to benefit not only head-injured service members in combat settings, but also civilian patients in resource-limited settings where there is no readily available CT scanner. Finally, exploring the performance of combinations of BBMs and imaging findings at various postinjury time points has the potential to identify and unlock the full potential of currently existing BBMs to contribute to a revised TBI classification system.

### Discovery and development of new BBMs

Investigative efforts should be encouraged to discover novel biomarkers with improved prognostic utility at acute time points and diagnostic utility at chronic time points. These promising candidate biomarkers should undergo thorough study in well-characterized and clinically representative cohorts using common data elements and predetermined sampling times based on current knowledge.

### Transparency, rigor, and reproducibility summary

A narrative review approach was used to summarize the evidence supporting the biomarkers for TBI, with a focus on primary research articles estimating their diagnostic and prognostic utility. The working group, comprising TBI experts from various fields (neurology, neurosurgery, neuroscience, clinical chemistry, military medicine, critical care, emergency medicine, and biostatistics) and representing multiple countries (Finland, Italy, Netherlands, Sweden, and the United States), leveraged their content expertise to identify key biomarkers. The group met regularly in 2023 to develop recommendations and compiled supporting data in tabular format. The initial draft of the recommendations was circulated for feedback from other working groups and was posted on the NINDS website 2 weeks prior to an open meeting at NIH in January 2024. During the meeting, expert and public feedback was gathered, which was then incorporated into an updated report. This report was reviewed by the Steering Committee, and further revisions were made following multiple working group discussions. The final report reflects the integration of feedback and expert input.

All data extracted from individual studies, including biomarker types, diagnostic accuracy metrics, and patient characteristics are presented in the article tables and supplement. This includes the full list of studies included in the review and the extracted data. The review process was independently verified by the Steering Committee to ensure consistency and reliability.

All relevant data and materials, including extracted data, are publicly available via the article tables and supplementary files as well as through direct request to the corresponding author. Any requests for data or clarifications regarding the methodology will be responded to within a reasonable timeframe.
