# Supplementary material for: Blood-Based Biomarkers for Improved Characterization of Traumatic Brain Injury: Recommendations from the 2024 National Institute for Neurological Disorders and Stroke Traumatic Brain Injury Classification and Nomenclature Initiative Blood-Based Biomarkers Working Group
Source: J Neurotrauma. 2025 Jul 9;42(13-14):1065–85. doi: 10.1089/neu.2024.0581 (PMC12409121; doi:10.1089/neu.2024.0581)
Supplement: Supplementary Data [file neu.2024.0581_supplementary_data.docx]

**Biomarkers with utility at Chronic time points (>30 days)**

NfL serum/plasma levels measured at chronic time points demonstrated potential for predicting post-TBI cerebral atrophy and reduced microstructural integrity. NfL serum/plasma levels at 180 days post-injury forecast white matter volume loss from 180 days to 1 year^3,4^; at 1 year, NfL levels correlated with the rate of volume loss in the mid anterior corpus callosum from 1 to 2 years^3,4^. These findings were replicated in an independent cohort, which reported that NfL serum/plasma levels at 8 months post-injury predicted volume loss in the white matter over the subsequent 5 years^5^. At 8 months post-injury, NfL levels anticipated increased mean diffusivity, a metric of injury in diffusion tensor imaging (DTI), from 8 months to over 5 years^5^. These findings underscore the potential utility of NfL as a prognostic tool in assessing the trajectory of cerebral structural changes following TBI.

Serum/plasma GFAP measured at approximately 8 months after TBI was found to be elevated in a subset of TBI patients, and in some it increased over the subsequent 5 years. This pattern contrasts with that of NfL, which uniformly decreases from 8 to 80 months post injury^5^. Moreover, GFAP serum/plasma levels at 8 months correlated with imaging biomarkers of axonal integrity, such as fractional anisotropy and mean diffusivity in the white and grey matter^5^.

Tau is a microtubule-associated protein that stabilizes microtubules in neurons and other cells. It has been widely studied as a biomarker of neurodegeneration, as it constitutes the core of neurofibrillary tangles, a main pathological feature in tauopathies. Tau undergoes complex post-translational modifications. Hyperphosphorylated Tau is prone to aggregation, making it a potential mediator of secondary neurotoxicity. Highly sensitive commercially available assays for p-Tau isoforms have been widely studied in neurodegenerative diseases.^6-8^. p-Tau isoforms shows promise in predicting global functional outcome and persistent symptoms 6-12 months following injury. In TBI patients with GCS scores ranging from 3-12, mean p-Tau231 levels in serum/plasma measured 1-6 months post-injury were moderately predictive of unfavorable 12-month GOS-E and Disability Rating Scale scores (15-30 vs. 0-4) with AUCs of 0.67 (p=0.03) and 0.71 (p=0.01), respectively^9^. Additionally, elevations in serum exosomal p-Tau181 levels among veterans with combat-related mild TBI, even several years post-injury (mean 7-11 years), exhibited significant correlations with post-traumatic and post-concussive symptoms^10^, highlighting the potential of p-Tau as a prognostic indicator for long-term outcomes and symptomatology in patients with TBI.

**References for Supplemental material**

1. Helmrich IRR, Czeiter E, Amrein K, et al. Incremental prognostic value of acute serum biomarkers for functional outcome after traumatic brain injury (CENTER-TBI): an observational cohort study. *The Lancet Neurology*. 2022;21(9):792-802.

2. Papa L, Ladde JG, O’Brien JF, et al. Evaluation of glial and neuronal blood biomarkers compared with clinical decision rules in assessing the need for computed tomography in patients with mild traumatic brain injury. *JAMA Network Open*. 2022;5(3):e221302-e221302.

3. Shahim P, Politis A, Van Der Merwe A, et al. Neurofilament light as a biomarker in traumatic brain injury. *Neurology*. 2020;95(6):e610-e622.

4. Shahim P, Politis A, Van Der Merwe A, et al. Time course and diagnostic utility of NfL, tau, GFAP, and UCH-L1 in subacute and chronic TBI. *Neurology*. 2020;95(6):e623-e636.

5. Newcombe VF, Ashton NJ, Posti JP, et al. Post-acute blood biomarkers and disease progression in traumatic brain injury. *Brain*. 2022;145(6):2064-2076.

6. Iqbal K, Liu F, Gong C-X. Tau and neurodegenerative disease: the story so far. *Nature reviews neurology*. 2016;12(1):15-27.

7. Thijssen EH, La Joie R, Strom A, et al. Plasma phosphorylated tau 217 and phosphorylated tau 181 as biomarkers in Alzheimer's disease and frontotemporal lobar degeneration: a retrospective diagnostic performance study. *The Lancet Neurology*. 2021;20(9):739-752.

8. Palmqvist S, Tideman P, Cullen N, et al. Prediction of future Alzheimer’s disease dementia using plasma phospho-tau combined with other accessible measures. *Nat Med*. 2021;27(6):1034-1042.

9. Rubenstein R, McQuillan L, Wang KK, et al. Temporal Profiles of P-Tau, T-Tau, and P-Tau: Tau Ratios in Cerebrospinal Fluid and Blood from Moderate-Severe Traumatic Brain Injury Patients and Relationship to 6–12 Month Global Outcomes. *Journal of Neurotrauma*. 2023;

10. Kenney K, Qu B-X, Lai C, et al. Higher exosomal phosphorylated tau and total tau among veterans with combat-related repetitive chronic mild traumatic brain injury. *Brain injury*. 2018;32(10):1276-1284.
