# Supplementary material for: Blood-Based Biomarkers for Improved Characterization of Traumatic Brain Injury: Recommendations from the 2024 National Institute for Neurological Disorders and Stroke Traumatic Brain Injury Classification and Nomenclature Initiative Blood-Based Biomarkers Working Group
Source: J Neurotrauma. 2025 Jul 9;42(13-14):1065–85. doi: 10.1089/neu.2024.0581 (PMC12409121; doi:10.1089/neu.2024.0581)
Supplement: Supplementary Table S1 [file neu.2024.0581_supplementary_tables1.docx]

| **Supplemental Table 1:** Incremental prognostic value of acute levels of GFAP, UCH-L1 and S100B over demographic, clinical, and radiological characteristics | | | | | |
| --- | --- | --- | --- | --- | --- |
|  | **TBI Patient Group**  [post injury timepoint] | **Outcome predicted** | **Incremental prognostic value** | | **Reference** |
|  |  |  | **∆AUC** (95% CI) | **∆R^2^** (95% CI) |  |
| **GFAP & UCH-L1** | |  |  |  |  |
|  | GCS 13-15  [0-24 hrs] | Mortality at 6mo | **0.004** (-0.006, 0.017) GFAP  **0.015** (0.001, 0.031) UCH-L1  **0.016** (0.001, 0.035) GFAP+UCH-L1 | **1.1%** (-1.9, 4.7%) GFAP  **3.7%** (-0.03, 7.9%) UCH-L1  **4.0%** (-0.5, 8.8%) GFAP+UCH-L1 | Helmrich (2022)^1^ |
|  |  | Unfavorable outcome (GOSE<4) at 6mo | **0.012** (0.003, 0.023) GFAP  **0.019** (0.006, 0.035) UCH-L1  **0.019** (0.006, 0.034) GFAP+UCH-L1 | **2.8%** (0.08, 5.4%) GFAP  **5.1%** (2.4, 8.3%) UCH-L1  **4.9%** (2.2, 8.2%) GFAP+UCH-L1 |  |
|  |  | Incomplete recovery (GOSE<8) at 6mo | **-0.002** (-0.006, 0.004) GFAP  **0.004** (-0.003, 0.015) UCH-L1  **0.003** (-0.005, 0.014) GFAP+UCH-L1 | **0.0%** (-0.7, 0.9%) GFAP  **1.2%** (0.0, 2.7%) UCH-L1  **1.0%** (-0.3, 2.6%) GFAP+UCH-L1 |  |
|  | GCS 3-12  [0-24 hrs] | Mortality at 6mo | **0.014** (0.005, 0.026) GFAP  **0.019** (0.008, 0.035) UCH-L1  **0.021** (0.009, 0.036) GFAP+UCH-L1 | **3.5%** (1.1, 6.7%) GFAP  **5.2%** (2.1, 9.1%) UCH-L1  **5.6%** (2.4, 9.5%) GFAP+UCH-L1 |  |
|  |  | Unfavorable outcome (GOSE<4) at 6mo | **0.021** (0.009, 0.036) GFAP  **0.031** (0.016, 0.048) UCH-L1  **0.030** (0.015, 0.048) GFAP+UCH-L1 | **4.4%** (1.8, 7.7%) GFAP  **6.6%** (3.3, 10.2%) UCH-L1  **6.4%** (3.2, 10.1%) GFAP+UCH-L1 |  |
|  |  | Incomplete recovery (GOSE<8) at 6mo | **0.014** (0.003, 0.031) GFAP  **0.019** (0.004, 0.040) UCH-L1  **0.020** (0.004, 0.041) GFAP+UCH-L1 | **2.1%** (0.5, 4.5%) GFAP  **3.1%** (0.8, 5.9%) UCH-L1  **3.1%** (0.8, 6.0%) GFAP+UCH-L1 |  |
| **S100B** |  |  |  |  |  |
|  | GCS 13-15  [0-24 hrs] | Mortality at 6mo | **0.001** (-0.011, 0.014) | **1.2%** (-1.5, 4.8%) |  |
|  |  | Unfavorable outcome (GOSE<4) at 6mo | **0.008** (-0.001, 0.019) | **2.0%** (0.01, 4.2%) |  |
|  |  | Incomplete recovery (GOSE<8) at 6mo | **0.006** (-0.002, 0.014) | **0.5%** (-0.5, 1.9%) |  |
|  | GCS 3-12  [0-24 hrs] | Mortality at 6mo | **0.025** (0.013, 0.042) | **5.7%** (2.4, 10.1%) |  |
|  |  | Unfavorable outcome (GOSE<4) at 6mo | **0.021** (0.010, 0.036) | **4.2%** (1.5, 7.4%) |  |
|  |  | Incomplete recovery (GOSE<8) at 6mo | **0.022** (0.008, 0.040) | **3.5%** (1.3, 6.1%) |  |
