# Supplementary material for: Blood-Based Biomarkers for Improved Characterization of Traumatic Brain Injury: Recommendations from the 2024 National Institute for Neurological Disorders and Stroke Traumatic Brain Injury Classification and Nomenclature Initiative Blood-Based Biomarkers Working Group
Source: J Neurotrauma. 2025 Jul 9;42(13-14):1065–85. doi: 10.1089/neu.2024.0581 (PMC12409121; doi:10.1089/neu.2024.0581)
Supplement: Supplementary Table S2 [file neu.2024.0581_supplementary_tables2.docx]

| **Supplemental Table 2.** Comparison of clinical decision rules and GFAP/UCH-L1 for prediction of traumatic abnormalities on head CT scan at acute timepoints^a^ | | | | | |
| --- | --- | --- | --- | --- | --- |
|  | **Sensitivity**  (95%CI) | **Specificity**  (95%CI) | **NPV**  (95%CI) | **PPV**  (95%CI) | **Reference** |
| GFAP/UCH-L1 | 1.00  (0.822-1.00) | 0.248  (0.203-0.300) | 1.00  (0.944-1.00) | 0.086  (0.056-0.128) | Papa  (2022)^2^ |
| Canadian CT Head Rule^b^ | 1.00  (0.821-1.00) | 0.331  (0.281-0.386) | 1.00  (0.957-1.00) | 0.095  (0.063-0.142) |  |
| New Orleans Criteria^c^ | 1.00  (0.822-1.00) | 0.160  (0.122-0.205) | 1.00  (0.914-1.00) | 0.077  (0.051-115) |  |
| National Emergency  X-Radiography Utilization Study II^b^ | 0.826  (0.604-0.943) | 0.521  (0.466-0.577) | 0.977  (0.938-0.993) | 0.109  (0.068-0.167) |  |
| Combination of GFAP/UCH-L1 & Canadian CT Head Rule^d^ | 1.00  (0.822-1.00) | 0.457  (0.402-0.513) | 1.00  (0.969-1.00) | 0.115  (0.076-0.169) |  |
| a. 349 patients with GCS 13-15, blood drawn within 4 hours of injury, 9.7% had traumatic intracranial injury (ICI) on head CT. b. For prediction of clinically significant traumatic ICI on head CT scan. c. For prediction of any traumatic ICI on head CT scan. d. GFAP alone equivalent to GFAP/UCH-L1 in combination. | | | | | |
